# Unlocking stroke prediction: Harnessing projection-based statistical feature extraction with ML algorithms

**DOI:** 10.1016/j.heliyon.2024.e27411

**Published:** 2024-03-06

**Authors:** Saad Sahriar, Sanjida Akther, Jannatul Mauya, Ruhul Amin, Md Shahajada Mia, Sabba Ruhi, Md Shamim Reza

**Affiliations:** a Deep Statistical Learning and Research Lab, Department of Statistics, Pabna University of Science & Technology, Pabna, 6600, Bangladesh; b Department of Statistics, Pabna University of Science & Technology, Pabna, 6600, Bangladesh

**Keywords:** Stroke, Risk prediction, Machine learning, PCA, FA, Medical diagnosis

## Abstract

Non-communicable diseases, such as cardiovascular disease, cancer, chronic respiratory diseases, and diabetes, are responsible for approximately 71% of all deaths worldwide. Stroke, a cerebrovascular disorder, is one of the leading contributors to this burden among the top three causes of death. Early recognition of symptoms can encourage a balanced lifestyle and provide essential information for stroke prediction. To identify a stroke patient and risk factors, machine learning (ML) is a key tool for physicians. Due to different data measurement scales and their probability distributional assumptions, ML-based algorithms struggle to detect risk factors. Furthermore, when dealing with risk factors with high-dimensional features, learning algorithms struggle with complexity. In this study, rigorous statistical tests are used to identify risk factors, and PCA-FA (Integration of Principal Components and Factors) and FPCA (Factor Based PCA) approaches are proposed for projecting suitable feature representations for improving learning algorithm performances. The study dataset consists of different clinical, lifestyle, and genetic attributes, allowing for a comprehensive analysis of potential risk factors associated with stroke, which contains 5110 patient records. Using significant test (*P*-value <0.05), chi-square and independent sample *t*-test identified age, heart_disease, hypertension, work_type, ever_married, bmi, and smoking_status as risk factors for stroke. To develop the predicting model with proposed feature extraction techniques, random forests approach provides the best results when utilizing the PCA-FA method. The best accuracy rate for this approach is 92.55%, while the AUC score is 98.15%. The prediction accuracy has increased from 2.19% to 19.03% compared to the existing work. Additionally, the prediction results is robustified and reproducible with a stacking ensemble-based classification algorithm. We also developed a web-based application to help doctors diagnose stroke risk based on the findings of this study, which could be used as an additional tool to help doctors diagnose.

## 1 Introduction

A stroke arises when bleeding or blood vessel congestion disrupts or hinders circulation to the brain, which causes the brain's cells and neurons to degenerate due to a lack of nutrients and oxygen [1]. The World Health Organization (WHO) ranks stroke as the second most prevalent cause of death worldwide [2]. Over a year, fifteen million people worldwide succumbed to strokes. Five million of these people perish, and another five million are permanently disabled, placing a strain on families and communities [3]. Stroke may affect anybody, from toddlers to adults; regardless, a handful of people are at higher levels of risk than others. Strokes expand steadily more common as individuals age; around two-thirds of strokes occur in people over the age of 65 [4]. Strokes can inflict various levels of harm on peripheral organs, including the heart, kidney, spleen, lung, and gastrointestinal system [5]. High blood pressure (hypertension), high cholesterol, type-2 diabetes, and those with a history of stroke, heart attack, or irregular heart rhythms such as atrial fibrillation are also risk factors for stroke [6]. Early predictions can mitigate the current death rate. So, all of these facts have led to the conduct of this research.

The implementation of machine learning in disease diagnosis has blossomed enormously in the earlier ten years [[7], [8], [9], [10], [11], [12]]. Accessible machine learning approaches have already been implemented in clinical applications for the rapid identification of stroke risk [[13], [14], [15]]. M. Kaur et al. [16] proposed a noninvasive approach using time series-based approaches such as LSTM, biLSTM, GRU, and FFNN to forecast strokes based on processed EEG data. The experimental results show that the GRU technique has the highest accuracy of 95.6%. N. Zafeiropoulos et al. [14] assessed the accuracy of several ML models based on diverse metric functions, comparing models such as SVM, KNN, LR, RF, XGB, and LGBM classifier. E. Dritsas et al. [17] conducted a study on predicting stroke risk through the application of various machine learning techniques. They utilized a stroke prediction dataset sourced from Kaggle, which originally consisted of 5110 observations. However, for their analysis, the researchers specifically selected 3254 observations. Their emphasis was solely on participants aged 18 and above, and eliminated the existing missing values from the original dataset. G. Sailasya et al. [18] similarly employed the Kaggle dataset to construct a predictive model for stroke prognosis. However, their study exclusively relied on various machine learning algorithms that are deemed insufficiently robust for accurate stroke prediction. Badriyah et al. [19] collected CT scan data from stroke patients at Hajj Hospital in Surabaya, Indonesia, with the aim of predicting stroke disease. The utilization of image data for stroke prediction is not consistently accessible, involves high costs, and can be time-consuming, posing challenges for swift diagnosis. K. Mridha et al. [13] assessed the efficacy of machine learning techniques in predicting strokes by employing the Kaggle stroke prediction dataset. The ML algorithm that demonstrated the highest performance among the models examined achieved an accuracy rate of approximately 91%, a result comparatively lower than our research. Kokkotis et al. [20] conducted a study to predict stroke disease. They used a dataset derived from Kaggle that contained 43,400 observations, which is not available now. The highest accuracy of this study was 73.52%, which is not enough for accurate stroke prediction.

Strategies for deep learning are being developed and optimized, which will substantially enhance the clinical application of machine learning technology. Additionally, the accuracy of stroke diagnosis and outcome prediction is constantly being improved by the implementation of new algorithms [21].

When features have several dimensions, the majority of ML algorithms struggle with complexity. Several feature selection and feature extraction strategies are employed to address this difficulty. Principal component analysis (PCA) and factor analysis (FA), both projection-based statistical methods, are beneficial for dimensionality reduction [22].

PCA works by transforming the original variables into a new set of uncorrelated variables, known as principal components, which capture the maximum variance in the data. These components are ordered by variance, with the first explaining the most variance. Without diminishing the number of feature information, this approach decreases the dimensionality of the dataset. The process of reducing dimensionality simplifies the dataset, aids in the visualization of the underlying structure, and provides more efficient analysis.

In FA, which is an extension of PCA, latent factors are used to show how variables are related to each other instead of basic components. Assuming that unobserved factors impact observable variables, its goal is to determine these components that account for shared variance between observed variables. By concentrating on these common elements, FA makes it possible to describe data more compactly by keeping the components that contribute the most to overall variation and eliminating the less important ones. This decreases the number of dimensions, allowing for a more interpretable and compact representation of the dataset, finding significant patterns and correlations while filtering out noise and extraneous information.

The ML technique and the projection-based dimension reduction approach perform poorly when the dataset is imbalanced [23]. In addition, the stroke prediction dataset reveals notable outliers, missing numbers, and a considerable imbalance across higher-class categories, with the negative class being larger than the positive class by more than twice. We investigated all previously disclosed data pre-processing approaches to enhance stroke risk patient prediction utilizing computer-aided diagnostic (CAD) methodologies.

In our research, we introduced the PCA-FA and FPCA methods for better representation of features that help enhance the accuracy of stroke disease prediction. We identified potential risk factors through rigorous statistical techniques. Additionally, we employed a stacking ensemble-based classification algorithm, combining SVM, RF, and XGBoost models, to construct the prediction model.

The proposed integrated technique enhances the accuracy of stroke risk categorization, avoids stroke misdiagnosis, and increases patient survival.

The comprehensive related study is presented in section 2. The stroke prediction data set is described in section 3. Section 4 studied several dimension reduction approaches. Section 5 includes a chapter on the proposed approach. The results and discussion are discussed in section 6. The conclusion is introduced in the last section.

## 2 Related works

In recent decades, the frequency of human diseases has increased compared to earlier times. When compared to other major illnesses, stroke diseases have a steadily increasing prevalence among those affected [24]. The scientific community has shown considerable interest in developing tools and processes for tracking and forecasting a wide range of illnesses that have a significant impact on human health. This section will review the most recent studies that use ML algorithms to predict the risk of stroke. Dritsas et al. [17] conducted a study on predicting stroke risk using various ML techniques. The research involved evaluating multiple models, including stacking, random forest (RF), and logistic regression (LR). Performance metrics such as accuracy, precision, recall, and AUC were used to assess the models. The findings emphasized the stacking model as the most effective, achieving an AUC of 98.9% and an accuracy of 98%. G. Sailasya et al. [18] utilized the Kaggle dataset to develop a predictive model for stroke prognosis. They utilized a range of learning techniques, including LR, decision tree (DT), RF, k-nearest neighbors (KNN), support vector machine (SVM), and naïve bayes (NB). Achieving an accuracy of 82% for stroke prediction, the NB algorithm outperformed the other algorithms. Minhaz et al. [25] obtained data from different hospitals in Bangladesh. They used ten ML algorithms and also proposed a method named ''weighted voting classifier''. The accuracy of the proposed study was 97%, with the weighted voting classifier outperforming the traditional classifiers. K. Mridha et al. [13] evaluated the effectiveness of ML techniques in stroke prediction, comparing six classifiers and applying explainable techniques like Shapley Additive Explanations (SHAP) and Local Interpretable Model-agnostic Explanations (LIME). The top-performing ML algorithm among the models under examination had an accuracy rate of about 91%, while the other models had accuracy rates between 83% and 91%. Badriyah et al. [19] gathered CT scan information from stroke patients at the Hajj Hospital in Surabaya, Indonesia. Eight ML techniques, including KNN, NB, LR, DT, RF, multi-layer perceptron (MLP-NN), deep learning, and SVM, are implemented to classify stroke diseases. Along with precision values (94.39%), recall values (96.12%), and F1-score (95.39%), RF produces the greatest degree of accuracy (95.97%). Kokkotis et al. [20] address the urgent need to use artificial intelligence (AI) as an appropriate tool to estimate the effect of numerous risk variables on the occurrence of strokes. Six well-known classifiers were compared to see how effective their proposed ML technique was, taking into account measures for prediction accuracy and generalization ability. The best accuracy (73.52%), specificity (73.43%), and AUC (83.30%) were obtained by the LR classifier. Additionally, they used SHAP to investigate how risk factors affected the outcome of the.

prediction. Tahia Tazin et al. [26] implemented four distinct models for accurate prediction using a variety of physiological indicators and ML methods, including LR, DT, RF, and voting classifier. With an accuracy of almost 96%, RF was the most accurate algorithm for this challenge.

However, certain researchers focused solely on specific age groups and opted to remove any prevailing missing values from the original dataset. Some researchers exclusively depended on machine learning algorithms deemed insufficiently robust for accurate stroke prediction. Furthermore, the use of image data for stroke prediction lacks consistent accessibility, entails high costs, and can be time-consuming, presenting challenges for prompt diagnosis. All these factors can be regarded as limitations in the existing body of work.

## 3 Dataset description and preprocessing

The dataset used in this study is sourced from Kaggle [27] and focuses on healthcare data related to strokes. It includes 5110 observations with eleven characteristics and one target output. Table 1 encompasses a detailed description of the proposed stroke dataset. Participants' information is included in the dataset, with each attribute being characterized as follows.

**Table 1:** Description of the stroke prediction dataset.

Attributes Name	Attributes Description	Attributes Type	Number of Observations	Missing Values	Outliers
age	Age of the patients	Integer	5110	0	Yes
gender	Gender (Male: 1, Female: 0)	Categorical	5110	0	No
heart_disease	Heart Disease (Yes: 1, No: 0)	Binary Integer	5110	0	No
hypertension	Hypertension (Yes: 1, No: 0)	Binary Integer	5110	0	No
work_type	Work Type (Govt_job: 0, Never_worked: 1, Private: 2, Self-employed: 3, children: 4)	Categorical	5110	0	No
ever_married	Marital Status (Yes: 1, No: 0)	Categorical	5110	0	No
Residence_type	Residence Area (Urban: 1, Rural:0)	Categorical	5110	0	No
avg_glucose_level	Average glucose level in blood	Integer	5110	0	Yes
bmi	Body Mass Index	Integer	4909	201	Yes
smoking_status	Smoking Status (Unknown: 0, formerly smoked: 1, never smoked: 2, smokes: 3)	Categorical	5110	0	No
stroke	Patient had a stroke (Yes: 1, No: 0)	Binary Integer	5110	0	No

**Fig. 1:**
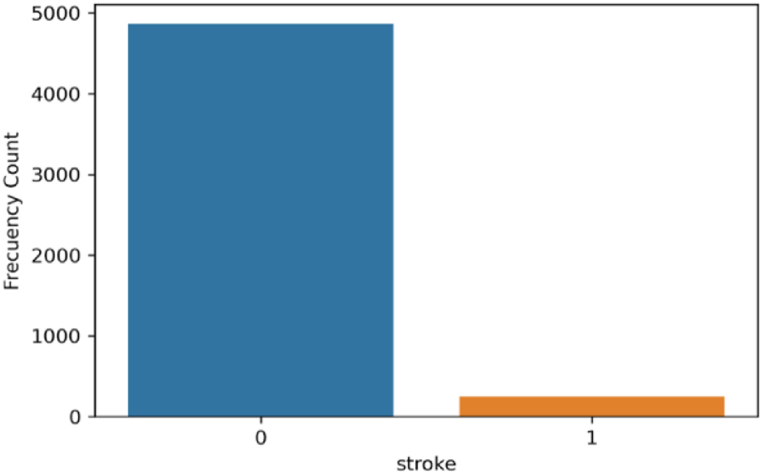
Diagram of patient class distribution.

**Fig. 2:**
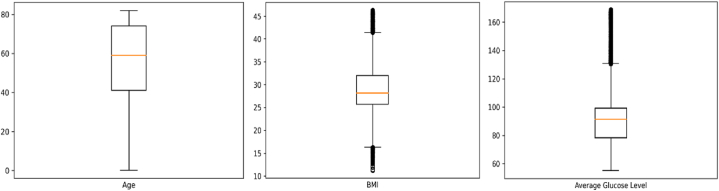
Boxplot of the variables in the dataset.

**Fig. 3:**
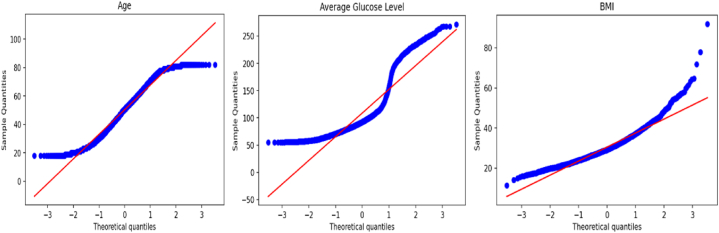
Q-Q plot of the dataset features.

Fig. 1 shows the bar diagram of the stroke patient class distribution. The target variable is highly imbalanced, with approximately 4.87% (249) having a stroke and 95.13% (4860) not having a stroke. The boxplot and interquartile range (IQR) approaches have been applied to detect outliers in this dataset. Fig. 2 illustrates the outliers in bmi and avg_glucose_level. To verify the normality of the dataset, we employed the Q-Q plot. We can infer from Fig. 3 that the age, bmi, and avg_glucose_level attributes are approximately normal. We have also confirmed the result by using the Shapiro-Wilk test and the Kolmogorov-Smirnov test.

Preprocessing is the first stage in data analytics, transforming raw data into a machine-readable format for analysis. It prepares data for subsequent stages, reduces processing time, and prevents errors. Before using algorithms, data should be processed since unprocessed data might yield objective findings. Preprocessing procedures include data cleansing, munging, normalization, reduction, and noise removal [[28], [29], [30]]. To avoid ambiguity and inaccurate results from mining, data cleansing and noise reduction are essential [31,32].

**Fig. 4:**
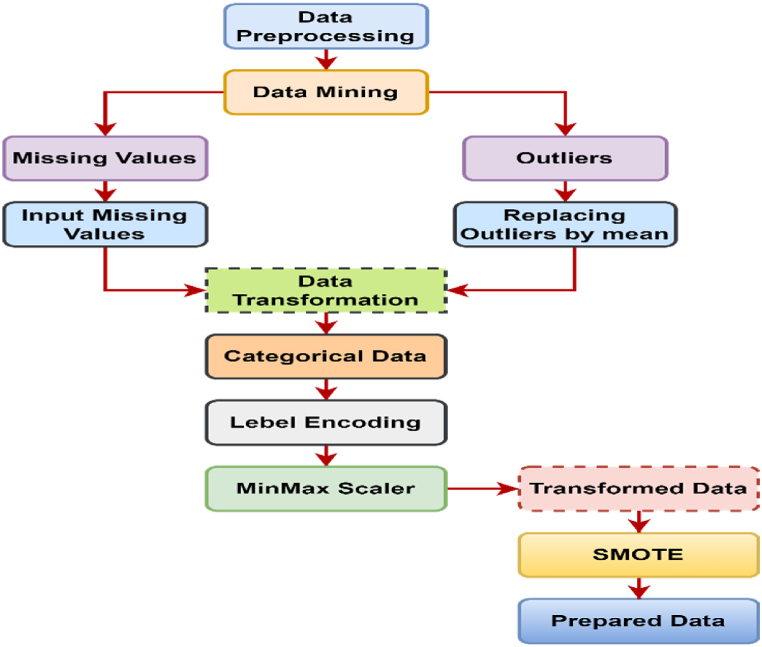
Data preprocessing diagram.

We pre-processed the original dataset to make it suitable for analysis. Initially, after detecting missing values, we replaced them with the median value, and outliers with the mean value. Categorical features are being transformed into numerical information using a label encoder. To ensure equitable representation of both stroke and non-stroke instances, class balancing was performed. We employed the synthetic minority over sampling technique (SMOTE) to oversample the minority class (stroke), which is displayed in Fig. 4. This method increased the dataset to 6150 instances, distributed equally between 3075 stroke patients and 3075 non-stroke cases.

## 4 Dimension reduction methods and algorithm

Dimensionality reduction is a technique for minimizing the number of random variables in a problem by finding a set of principal variables [33]. In many situations, a dataset has a significant number of input features, which complicates the process of predictive modelling. For training datasets with a large number of features, it is extremely challenging to visualize.

or predict the future; hence, dimensionality reduction techniques must be used [34]. Two approaches can be used: feature selection and feature extraction.

### 4.1 Feature extraction

Feature extraction includes lowering the dimensionality of a high-dimensional space to a lower-dimensional one. This approach is effective when there is a requirement to maintain critical information while boosting resource efficiency during data processing [35]. In predicting stroke patients, we performed PCA and FA to extract the most significant features from the stroke prediction dataset.

#### 4.1.1 Principal component analysis (PCA)

PCA is the most extensively used linear dimension reduction approach in machine learning, which is comparable to the method of keeping the error of the least square method in high-dimensional space [36]. The purpose of PCA is to reduce dimensions with as little loss as possible. To determine the most effective number of principal components (PCs) from the supplied data set, the cumulative explained variance technique is used [37].

Let us consider an arbitrary vector X,

$$X^{\prime }=\left[X_{1}X_{2}\cdots X_{U}\right]$$

Construct the matrix of variance and covariance,

$$\text{Var}\left(X\right)=\left[\begin{array}{cccc} \sigma_{1}^{2} & \sigma_{12} & \ldots & \sigma_{1u}\\ \sigma_{21} & \sigma_{2}^{2} & \ldots & \sigma_{2u}\\ \vdots & \vdots & \ddots & \vdots \\ \sigma_{u1} & \sigma_{u2} & \ldots & \sigma_{u}^{2} \end{array}\right]$$

Determine the variance-covariance matrix's eigenvalues $\left(\delta_{1},\delta_{2},\delta_{3},\ldots \ldots \delta_{u}\right)$ and eigenvectors $\left(w_{1},w_{2},w_{3},\ldots \ldots w_{u}\right)$. The number of primary components (u) is selected after ordering the eigenvalues. [38], i.e.

$$\begin{array}{c} Y_{1}=w_{11}X_{1}+w_{12}X_{2}+\cdots +w_{1u}X_{u}\\ Y_{2}=w_{21}X_{1}+w_{22}X_{2}+\cdots +w_{2u}X_{u}\\ Y_{p}=w_{u1}X_{1}+w_{u2}X_{2}+\cdots +w_{\text{uu}}X_{u} \end{array}$$

The largest variance among all the linear combinations is represented by the first PCA in this instance. The fraction of the overall variance owing to the $u^{\text{th}}$ main component is $\frac{\sum_{i=1}^{p}\delta_{i}}{\sum_{i=1}^{u}\delta_{i}}$ , where p < u.

Steps for PCA algorithm.

Step 1 Normalize the data: First step is to normalize the data that we have so that PCA works properly. This is done by calculating

$$Z=\frac{Value-mean}{Standard\;deviation}$$

Step 2 Calculate the covariance matrix: The covariance matrix is a square matrix, of U×U dimensions, where U stands for “dimensions”. It shows that pairwise feature correlation between each feature.

$$\text{Var}\left(X\right)=\left[\begin{array}{cccc} \sigma_{1}^{2} & \sigma_{12} & \ldots & \sigma_{1u}\\ \sigma_{21} & \sigma_{2}^{2} & \ldots & \sigma_{2u}\\ \vdots & \vdots & \ddots & \vdots \\ \sigma_{u1} & \sigma_{u2} & \ldots & \sigma_{u}^{2} \end{array}\right]$$

Step 3 Compute the eigenvalues & eigenvectors from the covariance matrix:

For eigenvalue, |A−δI|=0 and eigenvector, w(A−δI)=0where, I= Identity matrix

Step 4 Sorting the eigenvalues: We sort all the eigenvalues in decreasing order and simultaneously sort the eigenvectors accordingly in matrix p of eigenvalues, i.e.;

$$\delta_{1}\geq \delta_{2}\geq \ldots \geq \delta_{u}$$

Step 5 Calculating the new feature or principal components:

Proportion of total variance due to k-th principal component is,

$$\frac{\delta_{k}}{\delta_{1}+\delta_{2}+\ldots {+\delta }_{k}}\;;k=1,2,\ldots u$$

And the cumulative proportion of variance at k-th principal component is,

$$\frac{\delta_{1}+\delta_{2}+\ldots {+\delta }_{k}}{\delta_{1}+\delta_{2}+\ldots {+\delta }_{k}}\;;k<u$$

Step 6 We transform the original u dimensional data points into new k-dimensions and then just compute our samples onto the new subspace via the equation

$$Y=W^{\prime }X$$

$where\;W^{\prime }\;is\;the\;transpose\;of\;eigenvector\;matrix,W$

#### 4.1.2 Factor analysis (FA)

Factor analysis is a statistical technique employed to reduce a large set of variables into a smaller set of factors [39]. This method creates a common score by taking the most common variance out of all the factors. We can use this score as an index of all variables to do additional analysis [40]. Let us consider a feature vector X,

$$X^{\prime }=\left[X_{1}X_{2}\cdots X_{U}\right]$$

To calculate the mean vector μ for this random vector, perform the calculation

$$\mu^{\prime }=\left[\mu_{1}\mu_{2}\cdots \mu_{U}\right]$$

The m common factor obtained from the executed variable is:

$$\beta^{\prime }=\left[\beta_{1}\beta_{2}\cdots \beta_{U}\right]$$

Ultimately, the factor model will function as a multiple regression model, enabling the prediction of all u-observed variables [41].

$$\begin{array}{c} X_{1}=\mu_{1}+g_{11}\beta_{1}+g_{12}\beta_{2}+\cdots +g_{1m}\beta_{m}+\epsilon_{1}\\ X_{2}=\mu_{2}+g_{21}\beta_{1}+g_{22}\beta_{2}+\cdots +g_{2m}\beta_{m}+\epsilon_{2}\\ X_{u}=\mu_{u}+g_{n1}\beta_{1}+g_{n2}\beta_{2}+\cdots +g_{\text{nm}}\beta_{m}+\epsilon_{n} \end{array}$$

The general form of the matrix is,

X−μ=GB+ϵ

m<<U

$$\text{where},G_{ii}=\text{the}\;\text{loading}\;\text{of}\;\text{the}\;i^{\text{th}}\;\text{variable}\;\text{of}\;\text{the}\;j^{\text{th}}$$

In factor model observed variance-covariance matrix is expressed in terms of smaller number of factor and defined as

$$\Sigma =GG^{\prime }+\psi$$

That is the matrix of factor loadings times its transpose, plus a diagonal matrix containing the specific variances. The principal component method and maximum likelihood estimation are commonly used to estimate the parameters of a factor model. In the principal component method, we first determine the p eigenvalues along with eigenvectors for this variance-covariance matrix. The eigenvalues are $\left(\delta_{1},\delta_{2},\delta_{3},\ldots \ldots \delta_{u}\right)$ and the corresponding eigenvectors are $\left(e_{1},e_{2},e_{3},\ldots \ldots e_{u}\right)$. The following estimator for the factor loadings:

$${\hat{g}}_{ij}={\hat{e}}_{ij}\sqrt{{\hat{\delta }}_{j}}$$

ψ is now going to be equal to the variance-covariance matrix minus $GG^{\prime }$.

$$\hat{\psi }=\Sigma -GG^{\prime }$$

The total of the squared loadings for the $i^{th}$ variable is used to calculate the communalities ($h_{i}^{2})$ for that variable.

$${\hat{h}}_{i}^{2}=\sum_{j=1}^{m}{\hat{g}}_{ij}^{2}$$

We may use these results as multiple $R^{2}$ values for regression models predicting the important variables from the m factors. The communality for a given variable can be interpreted as the proportion of variation in that variable explained by the m factors. The cumulative explained variance approach is used to choose the optimal number of factors from the provided data set.

### 4.2 Feature selection

Feature selection involves selecting a subset of features from a larger collection based on specific criteria. The objective is to recognize and retain only the essential characteristics in a dataset, thereby eliminating redundant and superfluous ones [42]. This approach can reduce data processing complexity and enhance efficiency. We employ three distinct feature selection techniques: chi-square, recursive feature elimination (RFE), and a random forest classifier.

#### 4.2.1 Chi-square (${\;\chi }^{2})$

A chi-square (${\;\chi }^{2}$) statistic is a measure of the difference between the observed and expected frequencies of the outcomes of a set of events or variables. Chi-square is helpful for assessing such disparities in category data, particularly nominal variables. ${\;\chi }^{2}$ can be used to test whether two variables are related or independent from each other [43].

$$\chi^{2}=\sum \frac{{\left(O_{i}-E_{i}\right)}^{2}}{E_{i}}$$

where,O=Observedvalue(s),E=Expectedvalue(s)

#### 4.2.2 Recursive feature elimination

Recursive feature elimination (RFE) is a feature selection method that lessens the complexity of a model by selecting important characteristics and eliminating the less important ones. The selection procedure gradually eliminates each of these less important attributes until it reaches the ideal number required to ensure optimal performance. By using the model's "coef" or "feature importances" attributes, RFE ranks features [44]. After that, it recursively removes few of them of features per loop, eliminating any dependencies and collinearities that may have existed in the model. RFE reduces the number of features, increasing model effectiveness in the process.

#### 4.2.3 Random forest

Random forest (RF) consists of an amalgam of decision-trees. Combining the bootstrap aggregating approach and randomization in the selection of data nodes during the construction of a decision tree enhances the classification performance of a single tree classifier [45]. The feature space is divided into C regions $R_{c}$ using a decision tree with C leaves, 1 ≤ c ≤ C. For each tree, the prediction function f(y) is defined as:

$$f\left(y\right)={\left(y+a\right)}^{n}=\sum_{p=0}^{n}k_{c}\prod \left(\;y,R_{c}\right)$$

where C is the number of regions in the feature space, $R_{c}$ is a region appropriate to c; $k_{c}$ is a constant suitable to c:

$$\prod \left(\;y,R_{c}\right)=\left\{ \begin{array}{c} 1,\text{if}\;y\;\epsilon \;R_{c}\\ 0,\text{otherwise} \end{array}\right.$$

The final classification assessment is reached using the majority vote of all trees.

**Fig. 5:**
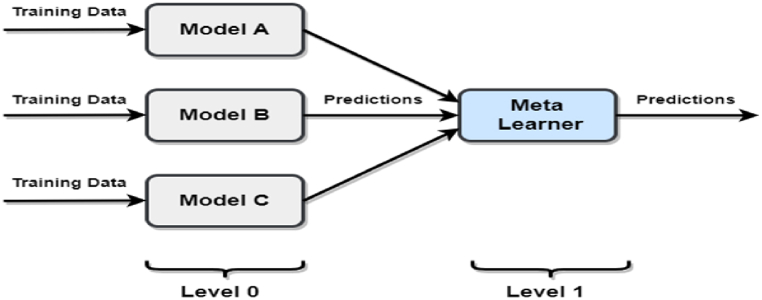
Stacking ensemble model.

Our aim is to identify the most crucial features within our dataset. The aforementioned techniques apply to produce the most potential subset of the feature for identifying stroke prediction. After getting the feature set, we fit different types of algorithms like SVM, LR, RF, etc. In this study, we used the stacking-based ensemble method. Fig. 5 provides an overview of the stacking ensemble model technique.

### 4.3 Stacking ensemble

One of the most popular and effective ensemble strategies in the world of machine learning is stacking. It distributes weights to the ML algorithms, which contain two layers of models-ground models and meta models. As a result, stacking typically outperforms all other ensemble ML strategies [26]. The ground models in stacking consist of a number of ML methods, but there is also an additional layer of the model called meta models. This model will give the ground model various weights before doing the prediction task in stacking.

## 5 Proposed methodology

**Fig. 6:**
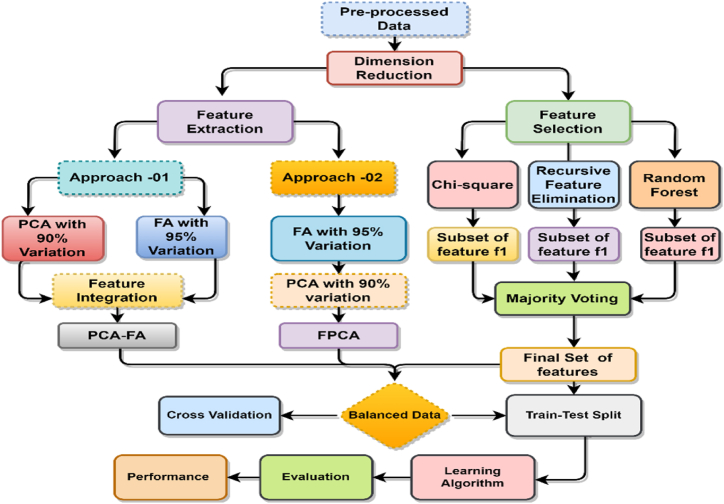
Illustration of the proposed model.

In the proposed methodology, we broadly classify it into two sections. Two feature extraction techniques, PCA-FA and FPCA, are suggested in the first section, and we identify the risk factors in the second section. The proposed methodology is shown in Fig. 6.

### 5.1 Proposed feature extraction approach: PCA-FA and FPCA

Fig. 6 illustrates the proposed feature extraction approaches, PCA-FA and FA. We have devised a PCA-FA strategy based on statistical projection (PCA and FA) to reduce feature dimensionality. This approach tries to identify relevant features and utilize all available methods to minimize the curse of dimensionality. The proposed feature extraction concept is fairly basic. First, PCA and FA are applied to the raw data. The PCs that can explain at least 95% of the total variation are kept, and the factors are organized using the cumulative explained variance method, which explains more than 95% of the variance. By employing 95% of the total variation in PCA, 9 principal components are extracted, while in the case of FA, extracting more than 95% of the variation results in 7 factors. Finally, both of the ordered extracted features of PCA and FA have been integrated.

In FPCA, we employ a two-step process: first, factor analysis (FA) is conducted on the training set, extracting 7 factors by considering more than 95% variation. Subsequently, the fitted and transformed factors are utilized to perform PCA on the test set, resulting in the extraction of 6 PCs using 95% variation. Here is the FPCA procedure into stepwise.

Step 1: Factor analysis on the training set

1. The observed variables in the training set are represented as a matrix X.
2. FA involves estimating the factor loadings (G) and obtaining factor scores (B) for the training set [46]:
  • The factor loadings matrix (G) is estimated using the equation: X=μ+GB+ϵ, where ϵ represents the specific or error terms.
  • The factor scores matrix (B) is calculated as: $B=G^{T}X$, where $G^{T}$ represents the transpose of the factor loadings matrix.
3. The factor loadings matrix (G) and factor scores matrix (B) obtained from FA on the training set are retained for further analysis.

Step 2: Transform the test set using the fitted factor loadings

1. The observed variables in the test set are represented as a matrix $X^{\prime }$.
2. The test set data is transformed into factor scores using the fitted factor loadings from the training set:
  • The factor scores matrix for the test set ($B^{\prime }$) is calculated as: ${B^{\prime }=G}^{T}X^{\prime }$, where $G^{T}$ represents the transpose of the factor loadings matrix obtained from the training set.

Step 3: Apply PCA on the transformed test set

1. PCA is performed on the transformed test set factor scores matrix ($B^{\prime }$).
2. The PCs that explain the maximum variance in the transformed test set are identified.
3. A subset of the PCs is retained based on criteria such as eigenvalues, scree plot, or a predetermined number of components, denoted as PC_subset.

### 5.2 Risk factor identification

**Fig. 7:**
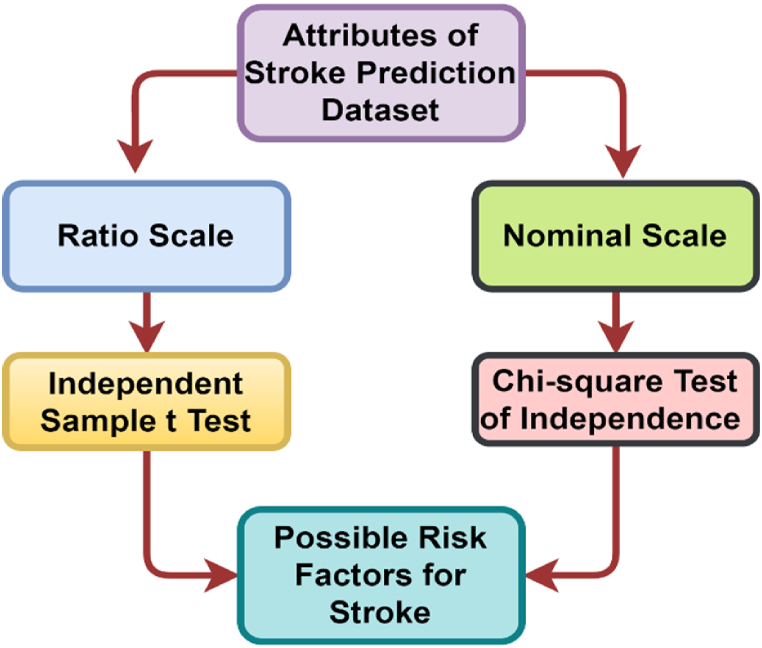
Risk factor identification diagram.

It is crucial to identify risk factors based on attributes. In our dataset, there are two types of attributes: the ratio scale and the nominal scale. Since different measures of scale exist, a single test alone may not yield appropriate results. To determine the risk factors, we employed a chi-square test for nominal scale attributes and an independent sample *t*-test for ratio scale attributes, which is illustrated in Fig. 7.

The factors that could potentially be connected to the risk factor in this dataset were identified using an independent sample *t*-test on ratio scale data. Here, $H_{0}\text{:}$ The mean of the stroke group and non-stroke group are equal, and $H_{1}$: The mean of the stroke group and non-stroke group are not equal. As can be apparent from Table 2, age and bmi have p-values of 0.000 and 0.000, respectively, indicating that they are associated with stroke, but avg_glucose_level has a p-value of 0.847, suggesting that it is not associated with stroke. The chi-square test has been carried out for nominal scale attributes to identify the risk factor for stroke. In this case, $H_{0}\text{:}$ There is no significant association between stroke and nominal attributes, and $H_{1}$: There is a significant association between both of them. As depicted in Table 2, heart_disease, hypertension, work_type, ever_married, and smoking_status are all associated with stroke since their p-values are all 0.000, but gender and Residence_type have p-values of 0.516 and 0.271, respectively, indicating that they are not associated with stroke. Therefore, age, heart_disease, hypertension, work_type, ever_married, bmi, and smoking_status have been identified as risk factors for stroke. After risk factor identification, we also proposed two feature extraction approaches.

**Table 2:** Attribute association test with stroke.

Attributes	Level of Measurement	Test statistic	*P*-value	Decision
age	Ratio Scale	t- statistic	0.000	Associated
gender	Nominal Scale	$\chi^{2}$ statistic	0.516	Not-associated
heart_disease	Nominal Scale	$\chi^{2}$ statistic	0.000	Associated
hypertension	Nominal Scale	$\chi^{2}$ statistic	0.000	Associated
work_type	Nominal Scale	$\chi^{2}$ statistic	0.000	Associated
ever_married	Nominal Scale	$\chi^{2}$ statistic	0.000	Associated
Residence_type	Nominal Scale	$\chi^{2}$ statistic	0.271	Not-associated
avg_glucose_level	Ratio Scale	t- statistic	0.847	Not-associated
bmi	Ratio Scale	t- statistic	0.000	Associated
smoking_status	Nominal Scale	$\chi^{2}$ statistic	0.000	Associated

## 6 Evaluation protocols and results discussion

The aim of this study is to compare the performance of various classification algorithms in order to identify the most accurate algorithm for predicting whether a patient will have a stroke or not. The suggested method's performance has been evaluated using the train-test split method. Using stratified random samples, the dataset is randomly divided into 70% training and 30% testing sets in an experimental strategy for evaluating the outcomes. The performance of various classifiers has been assessed and compared using the accuracy, precision, recall, F1-score, and receiver-operating curve (ROC). In addition, confusion matrix uses to indicate how well each classifier performs on the positive and negative classes separately, where two types give correct predictions and two types give wrong predictions for each classifier, including true positives (TP), true negatives (TN), false positives (FP), and false negatives (FN).

$$\text{Accuracy}=\frac{\text{TN}+\text{TP}}{\text{TN}+\text{TP}+\text{FN}+\text{FP}},\text{Precision}=\frac{\text{TP}}{\text{TP}+\text{FP}\;},\text{Recall}=\frac{\text{TP}}{\text{TP}+\text{FN}}$$

$$F1-\text{Score}=\frac{2*\text{Precision}*\text{Recall}}{\text{Precision}+\text{Recall}},\text{Misclassification}\;\text{Rate}=\frac{\text{FP}+\text{FN}}{\text{TN}+\text{TP}+\text{FN}+\text{FP}}$$

The performance of the classifiers using different methods such as PCA, FA, PCA-FA, FPCA, and feature selection is displayed in Table 3. Out of the 10 features, PCA shown that 9 PCs explained 95% of the data variation, with the RF classifier having the greatest accuracy 92.31%. In addition, precision 89.50%, F1-score 94.09%, and AUC 97.79% are also performed well. In factor analysis, seven factors are identified using the cumulative explained variance method, which explains more than 95% of the variance. The factors identified in the analysis are able to classify the data with an accuracy of 91.70% using the RF classifier.

**Table 3:** Comparison of the proposed methods' performance study.

Method	Classifiers	Accuracy (%)	Precision (%)	Recall (%)	F1-Score (%)	AUC (%)	Miss Rate (%)
PCA	LR	75.75	68.44	80.16	73.84	82.82	24.20
	**RF**	**92.31**	**89.50**	**94.84**	**92.09**	**97.79**	**7.69**
	KNN	88.75	80.65	96.23	87.76	93.95	11.20
	SVM	87.31	81.06	92.63	86.45	91.06	12.60
	GB	86.35	82.03	89.78	85.73	90.45	13.60
	XGB	91.52	87.58	95.08	91.18	96.07	8.48
FA	LR	78.80	73.52	82.20	77.62	86.96	21.20
	**RF**	**91.70**	**89.84**	**93.43**	**91.60**	**97.71**	**8.30**
	KNN	89.43	82.37	95.92	88.63	94.20	10.50
	SVM	83.88	77.64	88.71	82.80	91.80	16.10
	GB	84.80	79.83	88.65	84.01	91.61	15.20
	XGB	90.50	86.69	93.83	90.12	95.84	9.50
PCA-FA	LR	80.48	75.51	83.85	79.46	88.46	19.50
	**RF**	**92.55**	**90.53**	**94.35**	**92.40**	**98.15**	**7.45**
	KNN	89.54	82.16	96.37	88.70	94.16	10.40
	SVM	86.38	80.72	91.02	85.56	93.35	13.60
	GB	86.38	82.03	89.85	85.76	92.75	13.60
	XGB	91.52	87.58	95.08	91.18	97.37	8.48
FPCA	SVM	83.26	77.09	87.94	82.16	91.06	16.70
	RF	91.90	89.50	94.02	91.70	97.78	8.10
	XGB	90.02	87.01	93.44	89.61	96.07	9.98
	**Stacking**	**92.28**	**91.29**	**93.48**	**92.37**	**97.76**	**7.72**
Ensemble	SVM	90.36	85.18	95.02	89.83	95.21	9.64
Feature	RF	92.42	92.18	92.62	92.40	96.50	7.58
	XGB	94.78	94.37	95.15	94.76	98.78	5.58
	**Stacking**	**94.89**	**94.43**	**95.40**	**94.91**	**98.88**	**5.11**

The findings also demonstrate how effective the proposed PCA-FA approach is at classifying. It delivers the highest level of correct predictions among the studied algorithms using RF classifier, with an accuracy rate of 92.55%. The algorithm's ability to correctly identify positive cases within the dataset is shown by the precision score of 90.53%. The F1-score of 92.40%, which shows a balanced measure of precision and recall, further demonstrates the algorithm's ability to achieve high accuracy while accounting for both false positives and false negatives. The algorithm is also effective at distinguishing between positive and negative cases, as shown by the AUC score of 98.15%, and it has a good ability to rank instances appropriately. As an alternative technique, we have also proposed the FPCA strategy. The outputs are described in terms of accuracy, precision, recall, F1-score, AUC, and misclassification rate (miss rate). Among the classifiers, the stacking ensemble method achieves the highest performance, with an accuracy of 92.28%. It also demonstrates high precision, recall, F1-score, and AUC values, indicating its effectiveness in predicting stroke cases. The stacking ensemble has the lowest miss rate, further highlighting its capability in accurately identifying stroke instances. The RF classifier follows closely behind the stacking ensemble, achieving an accuracy of 91.90% and notable scores in other evaluation metrics. Similarly, the XGBoost classifier demonstrates a competitive performance with an accuracy of 90.02% and strong precision, recall, F1-score, and AUC values. In Table 3, we compared the performance of different statistical feature extraction methods and our proposed methods. According to the proposed PCA-FA, the RF classifier increased the stroke detection rate by 0.24–0.85% compared to PCA and FA features. In addition, precision, recall, F1, and AUC scores are increased by 1–2%. The final results of the research have been improved through the use of the PCA-FA technique, which achieved an accuracy of 92.55% and an AUC score of 98.15%, which is much better than the statistical feature extraction methods PCA and FA. In this research, the majority voting method provides six features, and the stacking ensemble approach outperforms all other classifiers when using the selected features. It achieves the highest accuracy 94.89%, precision 94.43%, recall 95.40%, and F1-score 94.91%.

**Fig. 8:**
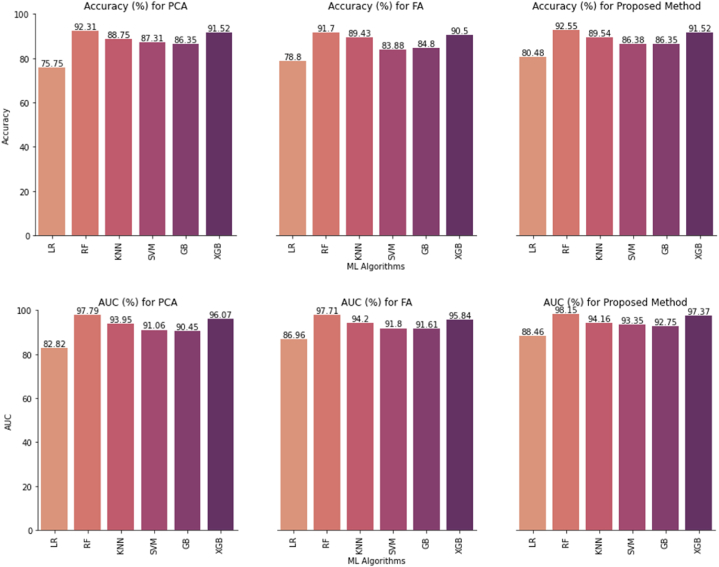
Model Accuracy and AUC comparison.

The stacking ensemble also demonstrates a high AUC value of 98.88%, indicating its ability to discriminate between stroke and non-stroke cases effectively. Furthermore, the stacking ensemble has the lowest miss rate of only 5.11%, indicating its effectiveness in correctly identifying stroke cases. Fig. 8 exhibits the accuracy and AUC scores of the different ML algorithms for the PCA, FA, and proposed methods.

**Fig. 9:**
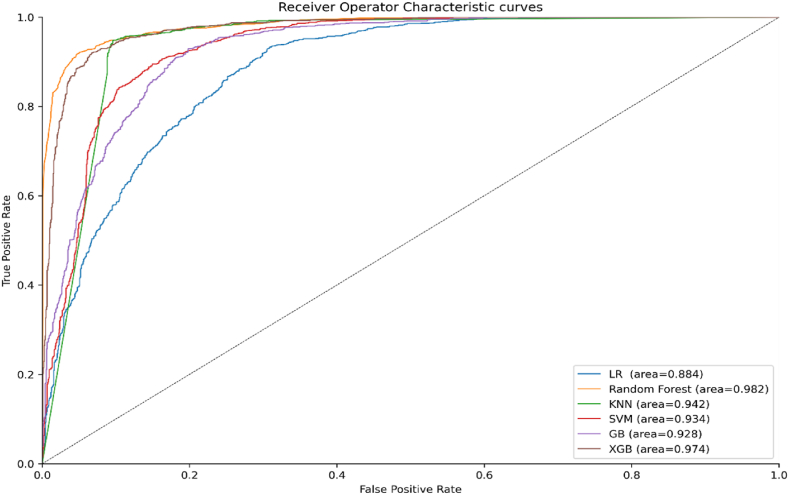
Roc curves for proposed PCA-FA method.

As our suggested method demonstrates in Fig. 9, the ROC curve for the RF classifier covers more than 98% of the area covered by the ROC curves for the other classifiers, demonstrating that the RF classifier is superior to the others. The proposed PCA-FA method and earlier research on stroke prediction utilizing a stroke prediction dataset are contrasted in Table 4. The results in Table 4 indicate that the proposed method outperforms the existing work, achieving the highest accuracy of 92.55% using the RF classifier for the stroke prediction dataset. In contrast to the findings of earlier research, G. Sailasya et al. [18] found a maximum accuracy of 82% using the NB classifier, which is 10.55% less accurate than the accuracy attained by our suggested technique.

**Table 4:** Comparison of the proposed method with the most relevant works.

Author & References	Classifiers	Accuracy (%)	Precision (%)	AUC (%)	Limitations
G. Sailasya et al. [18]	LR	78.00	77.50	–	a) Used limited ML algorithms that were not robust for accurate stroke prediction.
	DT	66.00	77.50	–	
	RF	73.00	72.00	–	
	K-NN	80.00	77.40	–	
	SVM	80.00	78.60	–	
	**NB**	**82.00**	**79.20**	–	
Krishna et al. [13]	**RF**	**90.36**	**88.00**	–	b) Lower accuracy and AUC score which is not enough for stroke prediction.
	LR	80.18	79.00	–	
	SVM	80.18	79.00	–	
	KNN	86.74	83.00	–	
	NB	76.03	74.00	–	
	XGB	89.02	88.00	–	
Kokkotis et al. [20]	**LR**	**73.52**	–	**83.30**	c) Focused on specific ages, removed prevailing missing dataset values.
	RF	71.19	–	81.24	
	XGboost	72.58	–	82.50	
	KNN	69.16	–	79.35	
	SVM	71.28	–	82.85	
	MLP	70.85	–	82.14	
PCA-FA	LR	80.48	75.51	88.46	
	**RF**	**92.55**	**90.53**	**98.15**	
	KNN	89.54	82.16	94.16	
	SVM	86.38	80.72	93.35	
	GB	86.38	82.03	92.75	
	XGB	91.52	87.58	97.37	

Another work by Krishna et al. [13] used an RF classifier and achieved a maximum accuracy of 90.36%, which is 2.19% less than our suggested approach. In contrast, Kokkotis et al. [20] used the LR classifier and obtained the best accuracy of 73.52%, which is much lower by 19.03% than our suggested approach. This highlights the significant improvement achieved by the proposed method compared to the previous approach, emphasizing the superior performance and effectiveness of the proposed PCA-FA model in stroke prediction.

**Fig. 10:**
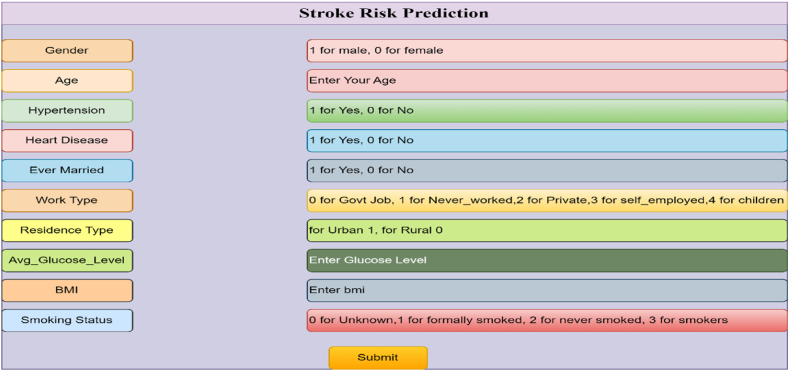
Input form in HTML code.

In order to implement the trained random forest classification model, a web application and flask application are developed. Flask application is a flexible ML framework that may be used for a variety of applications in the healthcare industry because of its ease of use, scalability, and interoperability with other frameworks. It is accessible to a larger range of people, such as end users, clinicians, and healthcare professionals, because of its adaptability and interoperability with several libraries. Data scientists and students are proficient in ML algorithms and libraries, but stakeholders may not understand the code in a business environment. That's why a ML web application has been created so that the model is accessible to them. The web application consists of a simple HTML code that includes an input form for users to enter parameter values for stroke prediction. When the user clicks the 'Submit' button, the entered parameters are passed to the flask application [18]. Fig. 10 provides a glimpse of the HTML page.

The flask application acts as a bridge between the web page and the trained ML model. It is a python code that receives the input values from the web page and forwards them to the model for prediction. The process flow is as follows: the user enters input values through the web page, the flask application receives these values, and then passes them to the trained model to make predictions. The implementation of the web application and flask application allows users to interact with the trained model through a user-friendly interface, facilitating the prediction of stroke occurrence based on the provided input parameters.

## 7 Conclusion

This research paper explores an enhanced integration strategy for stroke risk classification using statistical ML techniques. The study provides a comprehensive analysis of patients' attributes from health records for predicting stroke occurrence. For nominal scale and ratio scale attributes, we performed the chi-square test and an independent sample *t*-test to assess risk factors. The identified risk factors for stroke are age, heart_disease, hypertension, work_type, ever_married, bmi, and smoking_status. Furthermore, PCA and FA were conducted. The results reveal that a majority of principal components are necessary to explain a substantial variance in the dataset. Subsequently, different ML algorithms were employed using various feature sets and principal components. This paper also introduces two proposed methods: PCA-FA and FPCA. The prediction rate has increased between 0.24 and 0.85% in this study when the proposed strategy is applied to the stroke prediction dataset in comparison to PCA and FA features. Ratings are also boosted by 1%–2% for accuracy, recall, F1, and AUC. The final results of the research have demonstrated that the PCA-FA technique outperformed PCA and FA, achieving an accuracy of 92.55% and an AUC score of 98.15%. We have further used the feature selection approach, where the classifier's performance is slightly better than our proposed approach. However, the proposed strategy is helpful when we have a large amount of data and wish to limit the number of attributes without omitting any significant data. An application to facilitate efficient and rapid detection of this disease for healthcare professionals will be developed in the area of further research.

## Funding

This research did not receive any specific grant from any funding agency in the public, commercial or not-for-profit sector.

## Ethics declaration

Stroke prediction dataset, which is collected from the Kaggle repository, is a well-known secondary dataset. So, ethical approval is not required for this dataset.

## Dataset availability statement

For PIMA Indians diabetes dataset visit this URL (https://www.kaggle.com/datasets/fedesoriano/stroke-prediction-dataset).

## CRediT authorship contribution statement

**Saad Sahriar:** Writing – original draft, Formal analysis. **Jannatul Mauya:** Writing – review & editing, Conceptualization. **Ruhul Amin:** Writing – review & editing, Visualization, Investigation. **Md Shahajada Mia:** Software, Resources, Conceptualization. **Sabba Ruhi:** Writing – rev. **Md Shamim Reza:** iew & editing, Validation, Data curation, Writing – review & editing, Supervision, Investigation, Writing – review & editing, Supervision, Methodology, Conceptualization.

## Declaration of competing interest

The authors declare that they have no known competing financial interests or personal relationships that could have appeared to influence the work reported in this paper.

## References

[1] Goriely A. (Feb. 2015). Mechanics of the brain: perspectives, challenges, and opportunities. Biomech. Model. Mechanobiol. 2015 145.

[2] The top 10 causes of death.” https://www.who.int/news-room/fact-sheets/detail/the-top-10-causes-of-death (accessed January. 15, 2024).

[3] WHO EMRO | Introduction | Stroke, Cerebrovascular accident | Health topics.” https://www.emro.who.int/health-topics/stroke-cerebrovascular-accident/introduction.html (accessed December. 31, 2022).

[4] Hewitt J., Castilla Guerra L., Fernández-Moreno M.D.C., Sierra C. (2012). Diabetes and stroke prevention: a review. Stroke Res. Treat..

[5] Wang J. (2022). Peripheral organ injury after stroke. Front. Immunol..

[6] Boehme A.K., Esenwa C., V Elkind M.S., Fisher M., Iadecola C., Sacco R. (Feb. 2017). Stroke risk factors, genetics, and prevention. Circ. Res..

[7] Réda C., Kaufmann E., Delahaye-Duriez A. (2020). Machine learning applications in drug development. Comput. Struct. Biotechnol. J..

[8] Pasha S.J., Mohamed E.S. (2022). Advanced hybrid ensemble gain ratio feature selection model using machine learning for enhanced disease risk prediction. Inform. Med. Unlocked.

[9] Qezelbash-Chamak J., Badamchizadeh S., Eshghi K., Asadi Y. (2022). A survey of machine learning in kidney disease diagnosis. Mach. Learn. with Appl..

[10] Chang V., Bhavani V.R., Xu A.Q., Hossain M.A. (2022). An artificial intelligence model for heart disease detection using machine learning algorithms. Healthc. Anal..

[11] Pasha S.J., Mohamed E.S. (2020). Novel Feature Reduction (NFR) model with machine learning and data mining algorithms for effective disease risk prediction. IEEE Access.

[12] Mavrogiorgou A., Kiourtis A., Kleftakis S., Mavrogiorgos K., Zafeiropoulos N., Kyriazis D. (2022). A catalogue of machine learning algorithms for healthcare risk predictions. Sensors.

[13] Mridha K., Ghimire S., Shin J., Aran A., Uddin M.M., Mridha M.F. (2023). Automated stroke prediction using machine learning: an explainable and exploratory study with a web application for early intervention. IEEE Access.

[14] Zafeiropoulos N., Mavrogiorgou A., Kleftakis S., Mavrogiorgos K., Kiourtis A., Kyriazis D. (2023). Interpretable stroke risk prediction using machine learning algorithms. Lect. Notes Networks Syst..

[15] Campagnini S., Arienti C., Patrini M., Liuzzi P., Mannini A., Carrozza M.C. (2022). Machine learning methods for functional recovery prediction and prognosis in post-stroke rehabilitation: a systematic review. J. NeuroEng. Rehabil..

[16] Kaur M., Sakhare S.R., Wanjale K., Akter F. (2022). Early stroke prediction methods for prevention of strokes. Behav. Neurol..

[17] Dritsas E., Trigka M. (Jul. 2022). Stroke risk prediction with machine learning techniques. Sensors.

[18] Sailasya G., Kumari G.L.A. (2021). Analyzing the performance of stroke prediction using ML classification algorithms. Int. J. Adv. Comput. Sci. Appl..

[19] Badriyah T., Sakinah N., Syarif I., Syarif D.R. (2020). 2nd Int. Conf. Electr. Commun. Comput. Eng. ICECCE 2020.

[20] Kokkotis C. (2022). An explainable machine learning pipeline for stroke prediction on imbalanced data. Diagnostics.

[21] Mainali S., Darsie M.E., Smetana K.S. (2021). Machine learning in action: stroke diagnosis and outcome prediction. Front. Neurol..

[22] Picard M., Scott-Boyer M.P., Bodein A., Périn O., Droit A. (2021). Integration strategies of multi-omics data for machine learning analysis. Comput. Struct. Biotechnol. J..

[23] Shi H., Liu S., Chen J., Li X., Ma Q., Yu B. (2019). Predicting drug-target interactions using Lasso with random forest based on evolutionary information and chemical structure. Genomics.

[24] Dev S., Wang H., Nwosu C.S., Jain N., Veeravalli B., John D. (Nov. 2022). A predictive analytics approach for stroke prediction using machine learning and neural networks. Healthc. Anal..

[25] Emon M.U., Keya M.S., Meghla T.I., Rahman M.M., Al Mamun M.S., Kaiser M.S. (Nov. 2020). Proceedings of the 4th International Conference on Electronics, Communication and Aerospace Technology.

[26] Tazin T., Alam M.N., Dola N.N., Bari M.S., Bourouis S., Monirujjaman Khan M. (2021). Stroke disease detection and prediction using robust learning approaches. J. Healthc. Eng..

[27] Stroke Prediction Dataset | Kaggle.” https://www.kaggle.com/datasets/fedesoriano/stroke-prediction-dataset (accessed December. 25, 2022).

[28] Saroja S., Haseena S., Blessa Binolin Pepsi M. (2021). Data-driven decision making in IoT healthcare systems-COVID-19: a case study. Smart Healthc. Syst. Des. Secur. Priv. Asp..

[29] Kiourtis A., Mavrogiorgou A., Manias G., Kyriazis D. (2022). Ontology-Driven data cleaning towards lossless data compression. Stud. Health Technol. Inf..

[30] Ashfaq Z. (2022). Applied sciences Embedded AI-Based Digi-Healthcare. Appl. Sci..

[31] Mavrogiorgos K., Mavrogiorgou A., Kiourtis A., Zafeiropoulos N., Kleftakis S., Kyriazis D. (2022).

[32] Mavrogiorgos K., Kiourtis A., Mavrogiorgou A., Kleftakis S., Kyriazis D. (Jan. 2022). Int. Conf. Comput. Data Eng.

[33] Introduction to Dimensionality Reduction Technique - Javatpoint.” https://www.javatpoint.com/dimensionality-reduction-technique (accessed January. 4, 2023).

[34] Mweshi G. (Nov. 2019). Feature selection using genetic programming. Zambia ICT J..

[35] Introduction to Dimensionality Reduction Technique - Javatpoint.” https://www.javatpoint.com/dimensionality-reduction-technique (accessed July. 2, 2023).

[36] Tian J., Dong D., Liu Z., Wei J. (Jan. 2021). Key technologies and software platforms for radiomics. Radiomics Its Clin. Appl..

[37] 11.1 - Principal Component Analysis (PCA) Procedure | STAT 505.” https://online.stat.psu.edu/stat505/lesson/11/11.1 (accessed January. 6, 2023).

[38] Amin R., Yasmin R., Ruhi S., Rahman M.H., Reza M.S. (2023). Prediction of chronic liver disease patients using integrated projection based statistical feature extraction with machine learning algorithms. Inform. Med. Unlocked.

[39] Alkarkhi A.F.M., Alqaraghuli W.A.A. (Jan. 2019). Factor analysis. Easy Stat. Food Sci. with R.

[40] Factor Analysis - Statistics Solutions.” https://www.statisticssolutions.com/free-resources/directory-of-statistical-analyses/factor-analysis/(accessed December. 30, 2022).

[41] 12.1 - Notations and Terminology | STAT 505.” https://online.stat.psu.edu/stat505/lesson/12/12.1 (accessed December. 30, 2022).

[42] Cai J., Luo J., Wang S., Yang S. (Jul. 2018). Feature selection in machine learning: a new perspective. Neurocomputing.

[43] Bahassine S., Madani A., Al-Sarem M., Kissi M. (2020). Feature selection using an improved Chi-square for Arabic text classification. J. King Saud Univ. - Comput. Inf. Sci..

[44] Zeng X., Chen Y.W., Tao C., Van Alphen D. (2009). Feature selection using recursive feature elimination for handwritten digit recognition. IIH-MSP 2009 - 2009 5th Int. Conf. Intell. Inf. Hiding Multimed. Signal Process..

[45] Chen R.C., Dewi C., Huang S.W., Caraka R.E. (2020). Selecting critical features for data classification based on machine learning methods. J. Big Data.

[46] de Almeida F.A., Gomes G.F., Gaudêncio J.H.D., Gomes J.H. de F., de Paiva A.P. (Nov. 2019). A new multivariate approach based on weighted factor scores and confidence ellipses to precision evaluation of textured fiber bobbins measurement system. Precis. Eng..

